# Single-nucleotide polymorphism rs17548629 in RIPK1 gene may be associated with lung cancer in a young and middle-aged Han Chinese population

**DOI:** 10.1186/s12935-020-01215-w

**Published:** 2020-04-30

**Authors:** Shimin Yang, Fanglin Yu, Mingyan Lin, Linyi Sun, Junjie Wei, Cheng Lai, Lin Cai, Zhijian Hu, Fei He

**Affiliations:** 1 Clinical Medicine (Five-Year), Department of Clinical Medicine, Fujian Medical University, Fuzhou, 350122 Fujian People’s Republic of China; 2 Experiment Center, School of Public Health, Fujian Medical University, Fuzhou, 350122 Fujian People’s Republic of China; 3 Department of Epidemiology, School of Public Health, Fujian Medical University, Fuzhou, 350122 Fujian People’s Republic of China; 4 Fujian Provincial Key Laboratory of Environment Factors and Cancer, Fujian Medical University, Fuzhou, 350122 Fujian People’s Republic of China; 5 Fujian Provincial Key Laboratory of Tumor Microbiology, Fujian Medical University, Fuzhou, 350122 Fujian People’s Republic of China; 6 Key Laboratory of Ministry of Education for Gastrointestinal Cancer, Fujian Medical University, Fuzhou, 350122 Fujian People’s Republic of China

**Keywords:** Lung cancer, SNP, MicroRNA, *RIPK1*, rs17548629

## Abstract

**Background:**

Genetic biomarkers of lung cancer (LC) susceptibility may provide a basis for treatment and prevention. This study analyzed an association between SNPs (single nucleotide polymorphisms) in the complementary region of the 3′-UTR (3′ untranslated region) of microRNAs of the gene RIPK1 (receptor-interacting serine/threonine-protein kinase 1) and LC among an adult Han Chinese population aged younger than 60 years. Also explored the effect of regulation of the RIPK1 gene via rs17548629 and microRNA-1197 on the occurrence of LC.

**Methods:**

*RIPK1* variants (rs17548629, rs77736895) were determined in a population of 571 adults (younger than 60 years) with LC, and 609 gender- and age-matched healthy individuals. Bioinformatics methods predicted the microRNAs bound to rs17548629. Dual luciferase reporter assay was performed to confirm the presence of both rs17548629 and the predicted microRNA.

**Results:**

A mutation (T) of rs17548629 was associated with an increased risk for LC in this population under the codominant and recessive genetic models. The risk of lung adenocarcinoma in rs17548629 mutant carriers was 1.769-fold higher than that of the wildtype. In vitro, the luciferase activity of co-transfected mutant psiCHECK2-*RIPK1* and microRNA-1197 mimics was less than that of the group transfected with microRNA-1197 mimics only. Factorial analysis indicated interactions between microRNA-1197 mimics and genotypes of rs17548629.

**Conclusion:**

A mutation (T) of rs17548629 may increase the risk of LC/lung adenocarcinoma in adult Han populations younger than 60 years. When carrying the T allele, rs17548629 may be the target of hsa-miR-1197. This mutation may affect transcriptional level of the *RIPK1*, thereby promoting the occurrence of LC.

## Background

Lung cancer is extremely harmful to humans. According to the 2018 GLOBOCAN statistics, lung cancer accounts for 11.6% of all morbidity worldwide, and ranks first among all deaths due to cancer (18.4%) [1]. In China, lung cancer also has the highest morbidity and mortality. In 2015, the number of new cases and deaths were as high as 733,000 and 611,000, respectively [2], up to 80% of which were due to non-small cell lung cancer. Among the subtypes of non-small cell lung cancer, adenocarcinoma is more common than squamous cell carcinoma.

Lung cancer is often diagnosed at an advanced stage (stage III or IV) and its 5-year survival rate is only 15%. The risk of lung cancer increases significantly in people older than 40 years, with maximum morbidity and mortality rates at 75 years [3]. Therefore, it is highly important to explore the influencing factors and the mechanism of lung cancer.

Various factors increase the risk of lung cancer, including smoking and passive smoking [4–6], indoor environmental pollution [7], occupational exposure [8, 9], and psychological stress [10, 11], among others. Chronic inflammation of the lungs has also been proved associated with the development of lung cancer [12]. Persistent inflammation can induce malignant transformation of epithelial cells, creating an inflammatory microenvironment leading to oxidative stress, angiogenesis, and the inhibition of apoptosis, which further promotes the development of cancer.

The occurrence and progress of lung cancer is also regulated by genetics and epigenetics. Single nucleotide polymorphisms (SNPs) are commonly used as genetic markers in molecular epidemiological studies. SNP mutations are present in more than 1% of the population, and account for more than 90% of the variation in the human genome.

MicroRNAs are endogenous single-stranded small-molecule non-coding RNAs that are commonly found in eukaryotic cells. MicroRNAs regulate biological behaviors during the progression of lung cancer, such as cell proliferation, differentiation, and apoptosis [13, 14]. SNPs located in the 3′ untranslated region (UTR) of many genes may destroy the original microRNA binding site or generate new binding sites, and affect the post-transcriptional regulation of genes [13, 15]. All these factors participate in the pathogenesis of human disease.

The RIPK1 (receptor-interacting serine/threonine-protein kinase 1) protein can bind to TNFR1 (tumor necrosis factor receptor 1) protein, and subsequently recruit accessory factors (TRADD, LUBAC) which are situated downstream of the pathway, forming a complex. The complex promotes ubiquitylation of RIPK1, which activates NF-κB-related pathways to increase cell viability. RIPK1 can also can bind to FADD and caspase-8 leading to apoptosis, or bind to RIPK3 with subsequent programmed cell necrosis. Therefore, whether it leads cells to survive or die, RIPK1 has a significant regulatory role in the development of tumors. Some researchers have suggested that SNPs that are potential microRNA-binding targets in the 3′ UTR of the RIPK1 gene may be involved in the occurrence and progression of lung cancer [16].

The present study explored genetic biomarkers of lung cancer susceptibility that may provide a scientific basis for treatment and prevention. An initial case–control investigation was conducted of associations between SNPs in the 3′-UTR microRNA complementary region of the RIPK1 gene and lung cancer among a young and middle-aged Han Chinese population in Fujian province (adults aged younger than 60 years). Then, Bioinformatics methods were used to predict the microRNAs that can bind to rs17548629. Subsequently, an in vitro study determined the effect on protein levels of RIPK1 when wildtype or mutant genes of rs17548629 and the predicted microRNAs were introduced into 293T or A549 cells. The interaction of microRNA and SNP led to insight into the regulatory role of *RIPK1* in lung cancer.

## Materials and methods

### Participant populations

The case–control study comprised a case group of 571 adult patients aged younger than 60 years with newly diagnosed primary lung cancer, recruited between December 2006 and February 2014, and a control group of 609 gender- and age-matched healthy individuals (± 2 years) enrolled from July 2006 and February 2014. All the participants were residents of Fujian Province, living in Fujian for more than 10 years. The case and control groups were further stratified by age as ≤ 50, 51–54, and 55–59 years. In the present study, young and middle-aged was defined as an adult younger than 60 years.

Diagnoses of primary lung cancer were based on pathology after surgery or endoscopy. The patients were recruited from 3 hospitals: First Affiliated Hospital of Fujian Medical University; Fujian Medical University Union Hospital; and the Nine Hundredth Hospital of the People’s Liberation Army Joint Logistics Support Force. Individuals in the control group were randomly selected from the three hospitals and the community, but excluded the following: direct relatives of the patients, or with pulmonary inflammation, secondary lung cancer, a previous history of cancer, or other critical diseases.

### Data and sample collection

All epidemiological data were obtained through person-to-person interviews using a standardized questionnaire. The questionnaire covered general demographics; body mass index; food, tea, and alcohol consumption; living environment; smoking; passive smoking; history of lung diseases; occupational history; family history of cancer; and physical activity. All research variables were clearly defined.

Using the inquiry method for surveying dietary habits, respondents recalled their average frequency of consumption of foods (grams per day) in the last year (the year prior to study enrolment for all subjects) for a variety food items including seafood (fish, shellfish, snails, salted fish), fried food, fruits, vegetables, and salted vegetables. Questions regarding living environment included ventilation, use of exhaust fan, cooking oil fumes, irritating odors, and pollution in the area of residence. Smoking habits were defined as non-smoking, ≤ 30 pack-years (including smoking now and quit smoking now), and > 30 pack-years (including smoking now and quit smoking now). Drinking tea was defined as drinking at least 1 cup per week for more than half a year. Drinking alcohol was defined as drinking at least once a week for more than half a year. Physical activity included physical exercise (none, 1–3 ×/week, > 3×/week) and walking (none, occasionally, often).

A 5-mL non-fasting blood sample was collected from each participant for genotyping.

This study was approved by the Institutional Review Board of Fujian Medical University (Fuzhou, China) and all participants proved signed informed consent.

### Selection of SNPs and prediction of binding sites with microRNAs

The KEGG (Kyoto Encyclopedia of Genes and Genomes) database (http://www.genome.jp/kegg/) was used to retrieve the inflammatory signaling pathways downstream of RIPK1 related to lung cancer, and the node genes in the pathway were searched. Sequences and functional information of these genes were found using the *NCBI Gene Functional Annotation Database (http://www.ncbi.nlm.nih.gov/gene/).

SNPs at the target microRNAs on the 3′-UTR of the RIPK1 gene were predicted in reverse and screened using the following online prediction tools: microRNAanda (http:///www.microrna.org//); PicTar (http://pictar.mdc-berlin.de/); Diana-microT v 3.0 (http://diana.cslab.ece.ntua.gr/microT/); TargetScan Human 6.0 (http:///www.targetscan.org//); Target (PicTar (http://pictartar.mhttar.mdc.mdc-berlin.de/); Diana-MicroT v3.0 (http://diana.cslab.ece.ece.ntua.ntua.ntua.gr/://www.bioguo.org/microRNA SNP/).

Using the dbSNP (http://www.ncbi.nlm.nih.gov/SNP/) database, basic information regarding SNPs of the RIPK1 gene was obtained, including chromosome location, genome location, allele variation, risk allele, and minimum allele frequency. SNPs with minor allele frequency > 5% in the Han Chinese population were selected. RNAhybrid (http://bibiserv.techfak.uni-bielefeld.de/rnahybrid) was used to calculate the Gibbs free energy (G) difference (ΔΔG) between different alleles of SNPs and microRNAs, to evaluate the effects of these SNPs on the interaction between microRNAs and target genes. The greater the absolute difference (|ΔΔG|), the greater the influence of the SNP. Finally, the larger SNPs were selected.

Multiplex SNaPshot typing technology was used to achieve simultaneous automatic typing of the multiple SNP sites based on multiple primer extension. An SNP positively associated with lung cancer among the young and middle-aged Han population was selected as an experimental object of the present study.

The sequence information of the *RIPK1* 3′-UTR was downloaded from the University of California Santa Cruz website (http://genome.ucsc.edu/). The microRNAs and their target gene sequence variations were combined to predict the possible binding of microRNAs upstream and downstream of rs17548629 on the *RIPK1* 3′-UTR. The microRNA with the highest absolute context score value was selected as the experimental object.

### Luciferase reporter assay

The base sequence of the predicted microRNAs (hsa-mir-1197) combined with the SNP locus (rs17548629), was downloaded from the authoritative database for microRNAs (http://www.mirbase.org), i.e., 5′-UAGGACAUGGUCUACUUCU-3′. Using this, hsa-mir-1197 MIC (mimics) fragments were synthesized for subsequent cell transfection experiments.

To obtain the DNA fragment of the target gene, primers were designed by Oligo 7 software to amplify 51 base pair regions of rs17548629, wildtype (Wt) and mutant (Mut), on *RIPK1* 3′-UTR and hsa-mir-1197 inhibitor sponge sequences (Table 1). The resulting product solution was diluted 50-fold to a final concentration of 200 nM for the ligation reaction.

**Table 1:** Primer sequences

	Direction	Primer sequence
RIPK1 3′-UTR-Wt	+	TCGAGCTGGTACCTTCACCCAGCCTGAGTGCCCTGGAGAGGGAACAGGAAATGCTGGC
	–	GGCCGCCAGCATTTCCTGTTCCCTCTCCAGGGCACTCAGGCTGGGTGAAGGTACCAGC
RIPK1 3′-UTR-Mut	+	TCGAGCTGGTACCTTCACCCAGCCTGAGTGTCCTGGAGAGGGAACAGGAAATGCTGGC
	–	GGCCGCCAGCATTTCCTGTTCCCTCTCCAGGACACTCAGGCTGGGTGAAGGTACCAGC
Has-miR-1197 inhibitor sponge	+	TCGAGAGAAGTAGACCATGTGTCCTACGATAGAAGTAGACCATGTGTCCTATCACAGAAGTAGACCATGTGTCCTAGC
	–	GGCCGCTAGGACACATGGTCTACTTCTGTGATAGGACACATGGTCTACTTCTATCGTAGGACACATGGTCTACTTCTC

**Fig. 1:**
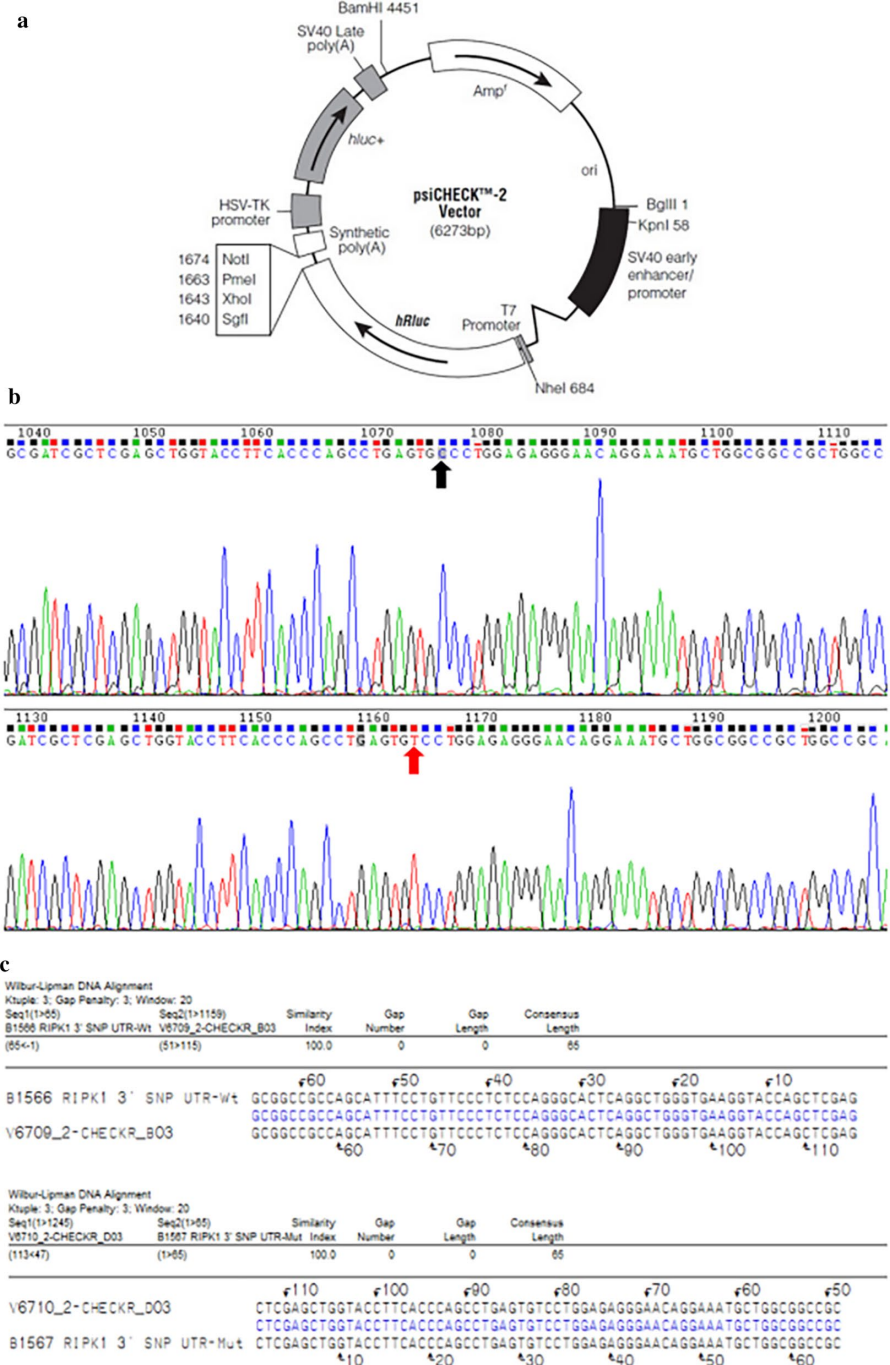
PsiCHECK2 vector plasmid map and sequence alignment of recombinant plasmids. **a** PsiCHECK2 vector plasmid used for gene over expression. This plasmid contains the kozak sequence, which can enhance gene expression. **b** Sequencing results of recombinant plasmids. Black arrows indicate wildtype of rs17548629 is “C” Allele. Red arrows show mutant of rs17548629 is “T” Allele. **c** Sequence alignment of recombinant plasmids and the gene fragment of interest

Carrier Psi-CHECK2 (Fig. 1a) was digested by SacI and XhoI at 37 °C for 2 h. The digestion system consisted of: 5 µL 10 × buffer, 5 µL Psi-CHECK2, 1 µL SacI, 1 µL XhoI, and 38 µL ddH2O. After agarose gel electrophoresis, the Psi-CHECK2 vector strip was recovered using a DNA gel Recovery Kit (Takara, Japan). Psi-CHECK2 bands obtained by double enzyme digestion, and the double-stranded template obtained by amplification, were linked using T4 DNA ligase to construct the recombinant plasmid for inserting into different target sequence fragments.

The plasmids were transferred into competent cells and evenly coated on Luria–Bertani broth plates containing 50 µg/mL ampicillin. The plasmids were cultured in a 37 °C incubator. After 16 h, 200 µL with positive clones were collected and sequenced. The sequencing results were compared with the target gene sequence, and the plasmids were extracted after confirmation.

Cells of the human renal epithelial cell line 293T and lung adenocarcinoma cell line A549 were cultured respectively in Dulbecco’s modified Eagle’s medium containing 10% fetal bovine serum (with 1.5 mML-glutamine, 100 U/mL penicillin, 100 μg/mL streptomycin), in an incubator with 37 °C ambient temperature and 5% CO_2_ saturated humidity. Cells in logarithmic growth phase were selected for the experiments.

The 293T cells and A549 cells were transiently transfected separately. Cells of each cell line were apportioned into the following 6 groups: positive control (psiCHECK2 vector inserted into a 1197 inhibitor sequence) and positive + miR-1197 mimics; wildtype psiCHECK2-*RIPK1* control and wildtype psiCHECK2-*RIPK1* + miR-1197 mimics; and mutant psiCHECK2-*RIPK1* control and mutant psiCHECK2-*RIPK1* + miR-1197 mimics. Cells were harvested 24 h after transfection and luciferase activity was measured using the Dual-Luciferase Reporter Assay System (Promega, Madison, Wisconsin, USA) in accordance with the manufacturer’s protocol. Experiments were repeated three times in triplicate.

The first pair (positive control without and with miR-1197 mimics) was used to exclude false negatives caused by experimental system problems. To observe whether the mimics bind to the allelic type plasmid, the controls (wildtype or mutant psiCHECK2-*RIPK1* without miR-1197 mimics) were used as blanks to the corresponding mimics group (wildtype or mutant psiCHECK2-*RIPK1* with miR-1197 mimics). The relative activity values of the groups wildtype psiCHECK2-*RIPK1* + miR-1197 mimics and mutant psiCHECK2-*RIPK1* + miR-1197 mimics were cross-referenced to determine if the Wt and Mut forms of SNPs differ with changes in luciferase activity.

### Statistical analysis

The Chi squared test (χ^2^) was used to compare the distribution differences between the case and control groups with regard to age, gender, education level, marital status, and other general demographic characteristics. The distribution of SNPs at a single site in the control group was analyzed to determine whether the distribution of SNP accorded with Hardy–Weinberg’s law of genetic balance. The SNP allele frequencies of the case and control groups were calculated, and the difference in allele frequencies was compared with the χ^2^ test. A binary unconditional logistic regression model was applied to analyze the association of each site with lung cancer among the study population under co-dominant, dominant, over-dominant, recessive, and additive genetic models. A multi-classification logistic regression model was used to evaluate the association of each site with histopathological types of lung cancer in the population under these genetic models. The odds ratios (ORs), adjusted ORs, and 95% confidence intervals (CI) were calculated.

For target gene validation, the *t* test was used to compare the relative activity of the reporter gene between the recombinant plasmid + mimics co-transfection groups and the corresponding control group. Analysis of variance of factorial design was applied to compare the activity of the reporter gene after the psiCHECK2 recombinant plasmid interacted with mimics. All the *P* values were bilateral test results, and the test level α was 0.05.

## Results

### General demographic characteristics of the subjects

In the case–control study, the case and control groups comprised 571 patients with lung cancer and 609 healthy individuals, respectively (Table 2). The 2 groups were statistically similar with regard to gender, age and marital status, but there was a significant difference in levels of education. Among the patients, there were 285 (49.9%) cases of adenocarcinoma, 140 (24.5%) squamous cell carcinomas, 47 (8.2%) small cell carcinoma, 39 (3.3%) alveolar cell carcinoma, 16 (2.8%) undifferentiated carcinoma, 14 (2.5%) large cell carcinoma, and 30 (5.3%) of other types of carcinoma.

**Table 2:** Demographics and pathologies of case and control groups, n (%)

	Case group	Control group	*χ*^2^	*p*
Age, years				
≤ 50	237 (41.5)	276 (45.3)	2.847	0.241
51–54	138 (24.2)	151 (24.8)		
55–59	196 (34.3)	182 (29.9)		
Gender				
Male	364 (63.7)	397 (65.2)	0.267	0.626
Female	207 (36.3)	212 (34.8)		
Education				
Elementary school or less	278 (48.7)	208 (34.2)	27.815	< 0.001
Middle school	246 (43.1)	319 (52.4)		
College and above	47 (8.2)	82 (13.5)		
Marital status				
Married	550 (96.3)	575 (94.4)	2.407	0.130
Unmarried and other	21 (3.7)	34 (5.6)		
Pathology				
Adenocarcinoma	285 (49.9)	–		
Squamous cell carcinoma	140 (24.5)	–		
Alveolar cell carcinoma	39 (3.3)	–		
Small cell carcinoma	47 (8.2)	–		
Large cell carcinoma	14 (2.5)	–		
Undifferentiated carcinoma	16 (2.8)	–		
Others	30 (5.3)	–		

### Association study between single SNP and susceptibility to lung cancer

The genotypes and allele distributions of rs17548629 and rs77736895 were not significantly different between the case and control groups (Table 3). The distribution of each SNP in the control group was in Hardy–Weinberg equilibrium, indicating that the subjects were representative of the general population. Associations were investigated between five genetic models (co-dominant, dominant, super-dominant, recessive, and additive) of *RIPK1* gene SNPs and primary lung cancers of different pathological types (Table 4).

**Table 3:** Genotypic and allelic distribution of each site in the case group and control group and Hardy–Weinberg genetic balance test in the control group

	Genotype/allele	Case group	Control group	*χ*^2^	*P*	Expected^a^	*χ*^2^
rs17548629	CC	309 (54.1)	323 (53)	3.931	0.139	327	0.816
	CT	210 (36.8)	247 (40.6)			238	
	TT	52 (9.1)	39 (6.4)			43	
	C	828 (72.5)	893 (73.3)	0.200	0.657		
	T	314 (27.5)	325 (26.7)				
rs77736895	CC	492 (86.2)	523 (85.9)	0.155	0.975	524	0.508
	CT	77 (13.5)	84 (13.8)			82	
	TT	2 (0.4)	2 (0.3)			3	
	C	1061 (92.9)	1130 (92.8)	0.020	0.901		
	T	81 (7.1)	88 (7.2)				

Data indicated as n (%), unless indicated otherwise

^a^Expected value of control

**Table 4:** Multivariate logistic regression analysis of SNPs

	Sort	Squamous cell carcinoma		Adenocarcinoma		Small cell carcinoma		Total lung cancer	
		Cs/cnt	OR (95% CI)^Δ^	Cs/cnt	OR (95% CI)^Δ^	Cs/cnt	OR (95% CI)^Δ^	Cs/cnt	OR (95% CI)^Δ^
rs17548629									
Codominant	CC	72/323	1.000	154/323	1.000	27/323	1.000	309/323	1.000
	CT	57/247	1.022 (0.666–1.567)	105/247	0.828 (0.598–1.146)	14/247	0.648 (0.321–1.307)	210/247	0.835 (0.634–1.099)
	TT	11/39	1.569 (0.704–3.496)	26/39	1.634 (0.915–2.919)	6/39	2.317 (0.822–6.532)	52/39	1.671 (1.010–2.766)*
Dominant	CC	72/323	1.000	154/323	1.000	27/323	1.000	309/323	1.000
	CT + TT	68/286	1.094 (0.728–1.645)	131/286	0.928 (0.683–1.262)	20/286	0.837 (0.445–1.577)	262/286	0.937 (0.722–1.216)
Super-dominant	CC + TT	83/362	1.000	180/362	1.000	33/362	1.000	361/362	1.000
	CT	57/247	0.968 (0.639–1.468)	105/247	0.778 (0.567–1.067)	14/247	0.580 (0.293–1.146)	210/247	0.782 (0.599–1.023)
Recessive	CC + CT	129/570	1.000	259/570	1.000	41/570	1.000	519/570	1.000
	TT	11/39	1.556 (0.713–3.394)	26/39	1.769 (1.007–3.109)*	6/39	2.734 (0.997–7.497)	52/39	1.803 (1.104–2.944)*
Additive			1.140 (0.828–1.569)		1.057 (0.830–1.346)		1.093 (0.668–1.788)		1.066 (0.869–1.308)
rs77736895									
Codominant	CC	122/523	1.000	252/523	1.000	39/523	1.000	492/523	1.000
	CT	17/84	0.938 (0.511–1.722)	33/84	0.822 (0.516–1.309)	8/84	1.397 (0.599–3.256)	77/84	1.000 (0.686–1.457)
	TT	1/2	2.650 (0.126–55.587)	0/2	–	0/2	–	2/2	1.446 (0.112–18.728)
Dominant	CC	122/523	1.000	252/523	1.000	39/523	1.000	492/523	1.000
	CT + TT	18/86	0.971 (0.535–1.762)	33/86	0.809 (0.509–1.288)	8/86	1.390 (0.597–3.239)	79/86	1.006 (0.692–1.463)
Super-dominant	CC + TT	123/525	1.000	252/525	1.000	39/525	1.000	494/525	1.000
	CT	17/84	0.934 (0.509–1.714)	33/84	0.824 (0.517–1.312)	8/84	1.400 (0.601–3.263)	77/84	0.999 (0.685–1.455)
Recessive	CT + CC	139/607	1.000	285/607	1.000	47/607	1.000	569/607	1.000
	TT	1/2	2.672 (0.127–56.085)	0/2	–	0/2	–	2/2	1.446 (0.112–18.719)
Additive			1.009 (0.570–1.784)		0.802 (0.509–1.266)		1.366 (0.595–3.133)		1.013 (0.704–1.458)

Cs/cnt, case/control

Co-dominant, TT VS CT VS CC; Dominant (CT + TT) VS CC; Super-dominant, (CC + TT) VS CT; Recessive, TT VS (CC + CT); Additive, TT VS CC

* *p* < 0.05

^Δ^ORs were adjusted for demographic characteristics (age, gender, marital status, and education level) and environmental factors (intake of food, drinking tea, alcohol consumption, living environment, smoking, passive smoking, history of lung diseases, occupational history, family history of cancer, body mass index and physical activity)

After adjustments for general demographic characteristics and environmental factors, under the co-dominant genetic model the risk of lung cancer in carriers with the *RIPK1* rs17548629 TT genotype was 1.671-fold (95% CI 1.01 to 2.766) that of the CC genotype carriers. Under the recessive genetic model, the risk of lung cancer in carriers of the *RIPK1* rs17548629 TT genotype was 1.803-fold (95% CI 1.104 to 2.944) that of the CC + CT genotype carriers. Rs77736895 was not associated with lung cancer susceptibility in this population, before and after adjusting for general demographic characteristics and environmental factors.

The multi-classification logistic regression analysis showed that rs17548629 was associated with a significantly increased risk of lung adenocarcinoma under the recessive genetic model (OR 1.769, 95% CI 1.007–3.109), but was not associated with an increased risk of other pathological types. Whatever the genetic model, there was no statistically significant association between rs77736895 and the respective pathological types of primary lung cancer.

### Bioinformatics analysis

The positive SNP site obtained from the above association studies was considered the research object, and the bioinformatics data were analyzed (Table 5). It was predicted that a new microRNA binding site would be generated on the 3′-UTR of *RIPK1* when the site was the T allele. There were three possible binding microRNAs. The hsa-microRNA-1197 with the largest change in absolute value of context score was selected as the experimental object.

**Table 5:** MicroRNAs that may bind near rs17548629 on the 3′-UTR of the RIPK1 gene

	Allele	miR-ID	miRSite	Contex score change
rs17548629	T	hsa-miR-1197	tgaGTGTCCTgga	− 0.049
		hsa-miR-4436a	tgagTGTCCTGga	− 0.018
		Hsa-miR-5000-3p	tgagTGTCCTGga	− 0.018

### Prediction of RIPK1 rs17548629 combined with hsa-mir-1197 regulating the development of lung cancer cells

The sequencing results of recombinant plasmids were consistent with those of the target gene sequence, indicating that the cloning direction was loaded correctly (Fig. 1b, c). In 293T cells, the luciferase activity of co-transfected mutant psiCHECK2-*RIPK1* and microRNA-1197 mimics was 87.5% that of the control group by transfection of microRNA-1197 mimics (P < 0.001). In A549 cells, the luciferase activity of co-transfected mutant psiCHECK2-*RIPK1* and microRNA-1197 mimics was 88.2% that of the control group by transfection of microRNA-1197 mimics (P = 0.001). This suggested that the rs17548629 T allele of the *RIPK1* gene was the target of hsa-mir-1197.

In 293T cells, the luciferase activity of co-transfected wildtype psiCHECK2-*RIPK1* and microRNA-1197 mimics was 95.3% that of the control group by transfection of microRNA-1197 mimics (P = 0.091). In A549 cells, the luciferase activity of co-transfected wildtype psiCHECK2-*RIPK1* and microRNA-1197 mimics was 94.6% that of the control group by transfection of microRNA-1197 mimics (P = 0.070). This suggested that *RIPK1* carrying the C allele in rs17548629 may not be the target of hsa-mir-1197.

**Fig. 2:**
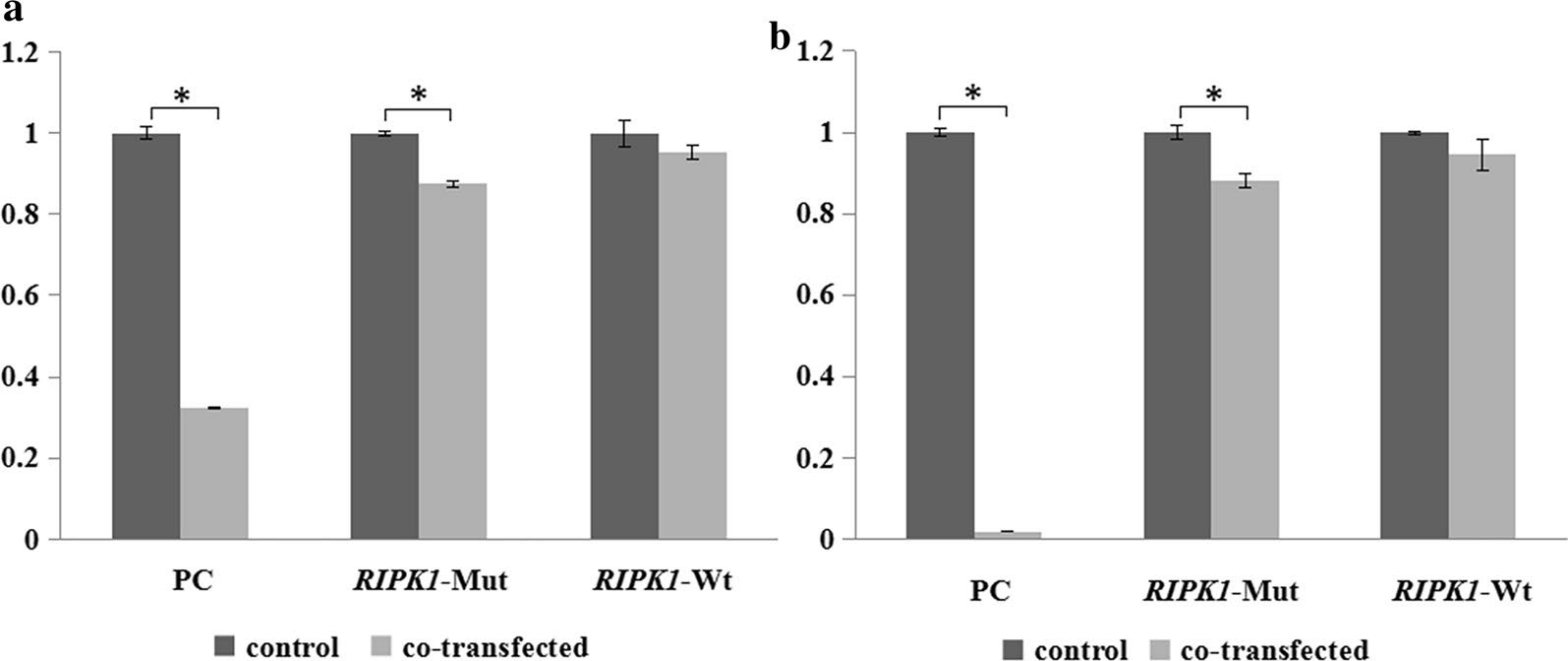
Detection results of relative activity of dual luciferase reporter gene. **a** Bar graph showing the relative fluorescence activity of the double luciferase reporter gene in 293T. **b** Bar graph showing the relative fluorescence activity of the double luciferase reporter gene in A549. Results are presented as the mean ± standard deviation. *P < 0.05

The factorial analysis showed that the relative fluorescence values of non-transfected and co-transfected mimics differed between the A549 and 293T cells. The relative fluorescence values of the genotypes at different sites were different, as was the presence or absence of mimics interacted with different genotypes at different sites (Fig. 2). The relative fluorescence activity in A549 and 293T cells of the co-transfected mutant psiCHECK2-*RIPK1*and microRNA-1197 mimics were 93.2% and 91.8% that of the co-transfected wildtype psiCHECK2-*RIPK1* and microRNA-1197 mimic carriers, respectively, and the relative fluorescence activity differences were statistically significant (Tables 6, 7). That is, when MiR-1197 mimics was co-transfected into A549 and 293T cells with mutant or wildtype psiCHECK2-*RIPK1*, the relative activities of the reporter gene differed in the rate of decrease. This prompted that the SNP may affect the binding strength of hsa-miR-1197 to RIPK1 in 293T. When there was a rs17548629 C > T mutation, a new binding site of hsa-miR-1197 was produced on the 3′-UTR of *RIPK1*, and the transcriptional level of the *RIPK1* decreased.

**Table 6:** Analysis of variance of mimics × genotypes in A549 and 293T cells

	Source of variation	SS	υ	MS	F	*p*
A549	Total variation	11.019	12	–	–	–
	Mimics	0.022	1	0.022	44.394	< 0.001
	Genotype	0.003	1	0.003	6.103	< 0.05
	Mimics × genotype	0.003	1	0.003	6.103	< 0.05
	Error	0.004	8	0.001	–	–
293T	Total variation	11.024	12	–	–	–
	Mimics	0.022	1	0.022	60.927	< 0.001
	Genotype	0.004	1	0.004	12.291	< 0.05
	Mimics × genotype	0.004	1	0.004	12.291	< 0.05
	Error	0.003	8	< 0.001	–	–

*SS* sum of squares of mean deviation, *MS* mean square

**Table 7:** Analysis of the effects of mimics × genotypes in A549 and 293T cells

	Genotype	Mean	SE	95% CI of the mean
A549	Wildtype	1	0.013	(0.975, 1.030)
	Mutant	1	0.013	(0.970, 1.030)
	Wildtype	0.946	0.011	(0.916, 0.976)
	Mutant	0.882	0.011	(0.852, 0.912)
293T	Wildtype	1	0.011	(0.975, 1.025)
	Mutant	1	0.011	(0.975, 1.025)
	Wildtype	0.953	0.011	(0.927, 0.978)
	Mutant	0.875	0.011	(0.850, 0.901)

*SE* standard error

## Discussion

This study found that a mutation (T) of *RIPK1* rs17548629 was associated with a significantly increased risk of lung cancer in an adult population aged younger than 60 years, under the codominant and recessive genetic model, after adjustments for general demographic characteristics and environmental factors. Moreover, the bioinformatics analysis revealed that hsa-miR-1197 may be combined with the polymorphic site of rs17548629, with a role in post-transcriptional regulation. Plasmids were constructed of wildtype and mutant target genes, and transfected into A549 and 293T cells. Changes in *RIPK1* transcriptional level in cells were observed with a dual luciferase reporter system. It was determined that rs17548629 is the target of hsa-miR-1197 when carrying the T allele. When a rs17548629 C > T mutation was present, a new binding site of hsa-miR-1197 was found in the 3′-UTR of *RIPK1*. The combination of the two was associated with low of *RIPK1* transcriptional level, and thereby may promote the progression of lung cancer.

Currently, the number of lung cancer patients under the age of 60 is gradually increasing, and the mortality rate shows a clear upward trend. According to data in the National Central Cancer Registry of China (NCCR), in the year 2015 the incidence and mortality rate of men ages 45 to 59 years and with lung cancer were 122.0/100,000 and 88.5/100,000 respectively; for women of the same age range, the rates were 53.9/100,000 and 32.5/100,000 [2]. Because young and middle-aged people are the working force of the nation, lung cancer in this age group will bring great trauma and economic pressures to society, as well as the patients and their families. Therefore the exploration of risk factors, mechanisms of lung cancer, and public health screening for biomarkers in this age group is very important.

The most common histological pathological type of lung cancer in young and middle-aged patients appears to be adenocarcinoma [17]. The present study found that, among all pathological types of lung cancer, only adenocarcinoma was associated with the polymorphism of *RIPK1* rs17548629 under the recessive genetic model. This possibility is real, but the number of cases is not sufficient to establish a strong statistical association in other pathological types.

RIPK1 was the first described member of the receptor-interacting protein (RIP) family, and has been a research hotspot in the field of cell signal transduction. RIPK1 participates in the conversion of apoptosis to necrotic apoptosis [18]. It is capable of interacting with the death receptor family member Fas and tumor necrosis factor receptor 1 (TNFR1). That is, it binds to the Fas-associated death domain (FADD) and the TNF receptor-associated death domain (TRADD) [19] to regulate the inflammatory response, apoptosis, and necrotic apoptosis signaling pathways. Tumor necrosis factor alpha-like (TNFα) is a potent pro-inflammatory cytokine that stimulates cells to cause the formation of a TNF-R1 signaling complex, to mediate downstream cell signaling. Activation of the TNF-R1 signaling complex is regulated by different types of ubiquitination, and may result in RIPK1-dependent and -independent cell death, including apoptosis and necrotic apoptosis [20].

Recently, RIPK1 has been proved to have anti-cancer effects, especially preventing proteasome degradation of TRAF2 to control the development of human liver cancer [21]. Yao et al. [22] studied the association between RIPK1 gene polymorphism and hepatocellular carcinoma. They found that the GG genotype of the SNP of *RIPK1* rs2272990 positively correlated with tumor-node-metastasis stage and lymph node metastasis of hepatocellular carcinoma, and negatively correlated with hepatic ischemia–reperfusion injury and prognosis of liver resection.

There have been few reports regarding associations between *RIPK1* or its polymorphisms and lung cancer. Because RIPK1 can affect tumorigenesis by regulating apoptosis, we speculate that the SNP of *RIPK1* is likely related to lung cancer. In the present study, rs17548629 and rs77736895 of *RIPK1* were selected, but the specific molecular mechanism remains unclear. After adjustments for general demographic characteristics and stable environmental factors, under the codominant genetic model the risk of lung cancer in carriers of the *RIPK1* rs17548629 TT genotype was 1.671-fold that of CC genotype carriers (95% CI 1.01 to 2.766). Under the recessive genetic model, the risks of lung cancer and lung adenocarcinoma of TT genotype carriers were, respectively, 1.803- and 1.769-fold that of CC + CT genotype carriers (95% CI 1.104 to 2.944 and 1.007 to 3.109). There was no correlation between rs77736895, another site of *RIPK1*, and lung cancer in this population.

According to the NCBI GenBank database (http://www.ncbi.nlm.nih.gov/snp), the global minor allele frequency of rs17548629 is 0.0931. However, the minor allele frequency of the Han Chinese is 0.268, which is higher than that of other ethnic groups. *RIPK1* is located in the 6p25.2 region of the chromosome, and the positive site rs17548629 screened in this study is located in the 3′-UTR region of *RIPK1*. The bioinformatics analysis predicted that rs17548629 C>T will generate a new hsa-miR-1197 binding site on the 3′-UTR of RIPK1, which inhibits the translation of RIPK1 mRNA.

Hsa-miR-1197 was isolated from neuroblastoma by Afanasyeva et al. [23] in 2008 and belongs to the miR-379 gene family. Its biological function is not clear. The present study found that the RIPK1 rs17548629 C>T mutation in young and middle-aged Han can significantly increase the risk of lung cancer and lung adenocarcinoma. Combined with the predictions of the present bioinformatics analysis, we speculate that this may be due to the interaction between has-miR-1197 and 3′-UTR. This interaction inhibits transcription of the RIPK1 gene, affects its inhibition of inflammation or induction of programmed apoptosis and necrotic apoptosis, and then leads to malignant progression of lung cancer. Yu et al. [24] used a combined priority method to predict new microRNA-disease associations by maximizing network information flow, with considerable effectiveness and stability. Then this method was applied to lung and breast tumors, predicting the top 30 microRNA candidates, which were consistent with the published literature and databases.

To investigate whether the rs17548629 mutation will generate a new hsa-miR-1197 binding site on the 3′-UTR of *RIPK1*, a dual luciferase reporter gene assay was conducted in the present study. The results obtained by the 293T and A549 cell groups were similar. Although the absolute value of the fluorescence value changed little, the RIPK1-Mut group statistically did bind to miR-1197 to a certain extent; the RIPK1-Wt group did not bind to miR-1197. These results were consistent with the bioinformatics prediction. The rs17548629 C>T variant did produce a new binding site for hsa-miR-1197 on the 3′-UTR of *RIPK1*, but the binding may be less tight. Thus, we hypothesize that when the concentration of miR-1197 and *RIPK1* mRNA is moderate, the rs17548629 C>T mutation increases the binding strength between them, and the protein levels of RIPK1 may decrease. Moreover, the inhibition of inflammation or induction of programmed apoptosis and necrotic apoptosis is weakened, leading to the development of lung cancer. To clarify the mechanism, it would be necessary to determine the concentration of miR-1197 in lung cancer cells. Western blot and real-time fluorescence quantitative PCR should be used to detect changes in protein and mRNA levels expressed by alleles of the target gene in lung cancer cells, after overexpression or inhibition of microRNA. Moreover, the ratio of miR-1197 and mRNA in lung cancer cells under natural conditions needs to be determined.

The present general population and cytology study preliminarily verified an association between SNP variants on RIPK1 rs17548629 and lung cancer. It provides new targets and ideas for the treatment of lung cancer and drug research, which has certain clinical significance. However, due to the multiplicity of RIPK1 protein functions, determining the specific molecular mechanism was beyond the scope of the present study. Moreover, the research was limited to the general population and the cytology, without in vivo validation. Therefore, generalized conclusions are not yet possible. However, the present results warrant experiments with animal models to confirm that SNP variants on RIPK1 rs17548629 are associated with the progression of lung cancer.

## Conclusion

*RIPK1* rs17548629 mutation (T) may increase the risk of lung cancer in young and middle-aged Han people under co-dominant and recessive genetic models, and increase the risk of lung adenocarcinoma in recessive genetic models. The results of the dual luciferase reporter assay showed that rs17548629 may be a target of hsa-miR-1197 when carrying the T allele. This mutation may affect transcriptional level of the RIPK1 gene via the interaction between hsa-miR-1197 and rs17548629 in the *RIPK1* 3′-UTR, thereby promoting the occurrence of lung cancer.

## Data Availability

The datasets used and analysed during the current study are available from the corresponding author on reasonable request.
